# Progressive multifocal leukoencephalopathy in a patient with mediastinal teratoma: a case report

**DOI:** 10.1186/s12883-022-02563-y

**Published:** 2022-01-27

**Authors:** Wei Wang, Hui Yang, Yueshan Piao, Meina Quan, Dongmei Guo

**Affiliations:** 1 Innovation Center for Neurological Disorders and Department of Neurology, Xuanwu Hospital, Capital Medical University, National Clinical Research Center for Geriatric Diseases, Beijing, China; 2 Department of Neurology, The Second Affiliated Hospital of Guizhou University of Traditional Chinese Medicine, Guiyang, China; 3 Department of Pathology, Xuanwu Hospital, Capital Medical University, Beijing, China

**Keywords:** Progressive multifocal leukoencephalopathy, Mediastinal teratoma, Lymphopenia, Mirtazapine, Case report

## Abstract

**Background:**

Progressive multifocal leukoencephalopathy (PML) is a rare demyelinating lytic brain infection caused by the John Cunningham virus (JCV). JCV manifests primarily in patients with innate immunodeficiency or taking immunomodulatory medications. In this case study, we report a PML patient with comorbid mediastinal teratoma and mild lymphopenia.

**Case presentation:**

A 73-year-old female presented with a 3-month history of progressive hemiplegia, hemianopsia, and cognitive impairment. She was diagnosed as PML by cerebrospinal fluid metagenomics sequencing and brain biopsy. Extensive immunological tests did not reveal an apparent immunodeficiency, but further work-up revealed that the PML was most likely the first presentation of mediastinal teratoma and the mild lymphopenia. Mirtazapine and immunoglobulin were started, the patient’s condition was relatively stable and approved to be discharged from hospital. But unfortunately, she died of the lung infection 10 months after first presentation.

**Conclusions:**

This case confirms that mediastinal teratoma may induce the lymphopenia and trigger PML, delayed or incorrect diagnosis may worsen the course of the disease and result in poor prognosis.

## Background

Progressive multifocal leukoencephalopathy (PML) is an opportunistic infection of white and gray matter cells caused by the John Cunningham virus (JCV) reactivation within the brain. JCV is present in 50–70% of the general population, while PML occurs in about 0.2/100,000 of the general population [1]. JCV is an opportunistic pathogen [2], the risk of PML is increased in patients with chronic inflammatory diseases or autoimmune diseases, even in the absence of treatments that induce an immunosuppressive status [3]. PML is a relevant topic in neurological fields and has been associated with therapeutic immunosuppression in patients with multiple sclerosis (MS) [4]. Various factors have been identified with increased risk of developing PML, including a positive JCV serology, natalizumab (NTZ) administration for > 2 years, and prior use of immunosuppressive agents [5]. Clinicians can employ such tools for patients’ risk stratification, but the incidence of PML among patients receiving NTZ therapy has not changed [5]. All above considered, a therapeutic switch in patients who respond to NTZ but are exposed to a high PML risk represents an important and increasingly frequent therapeutic challenge in MS clinical practice.

PML is also associated with lymphoproliferative disorders and patients often show first neurological symptoms [6]. There are recent reports of PML cases either with minimal immunodepression or even without any evidence of immunodepression [7, 8]. However, a comorbid PML and mediastinal teratoma has rarely been described, thus posing a particular diagnostic challenge. Here, we present PML which manifested spontaneously in a patient who had a diagnosis of asymptomatic mediastinal teratoma.

## Case presentation

**Fig. 1:**
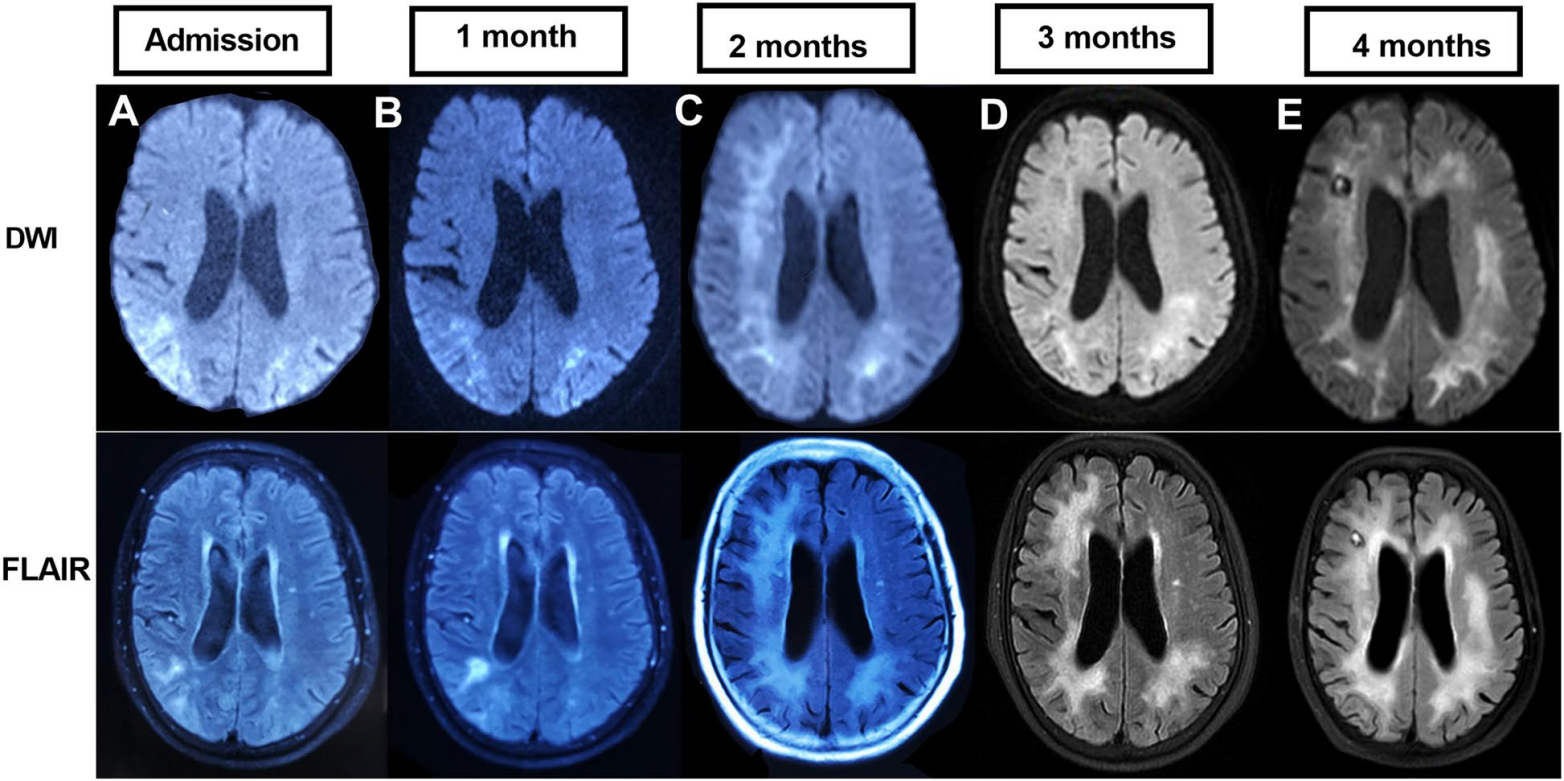
MRI shows hyperintense signal in DWI and FLAIR images. **A**-**E** (from the beginning of disease to 5 months) shows that the white matter lesions were gradually aggravated with the progress of the disease

A 73-year-old female patient was transferred to our neurological department after he had been admitted to another hospital twice before. The first admission had taken place 3 months before in march 2020 due to gradually worsening of hemiplegia and visual impairments (hemianopsia to the left). The MRI had shown Diffusion-weighted imaging (DWI) and T2-weighted-fluid attenuated inversion recovery (T2-FLAIR) hyperintensity in the right temporooccipital and frontal lobes that had been interpreted as an ischemic stroke. She was diagnosed as cerebral infarction and treated with antiplatelet and hypolipidemic drug. But the symptoms gradually worsened, cognitive impairment and hallucination emerged in May 2020. The MRI had displayed a massive lesion in both hemispheres predominantly affecting the right white matter (Fig. 1). The patient clinically deteriorated during the following weeks and was referred to our hospital for further work-up.

Neurologic examination, including an extensive neuropsychological workup, found cognitive impairment, a nonfluent aphasia, the decline of temporal and spatial orientations, attentional and executive defects, and left visual field defect. The muscle strength of the limbs was decreased. The muscle tension of limbs was normal, and the tendon reflex of limbs were normal. Bilateral Babinski signs are positive.

Her medical history mentioned under treatment for high blood pressure. She had an asymptomatic teratoma in the mediastinum (Fig. 3), which was discovered incidentally 22 years ago and has not changed since discovery. There was no history of immunosuppressant use.

**Fig. 2:**
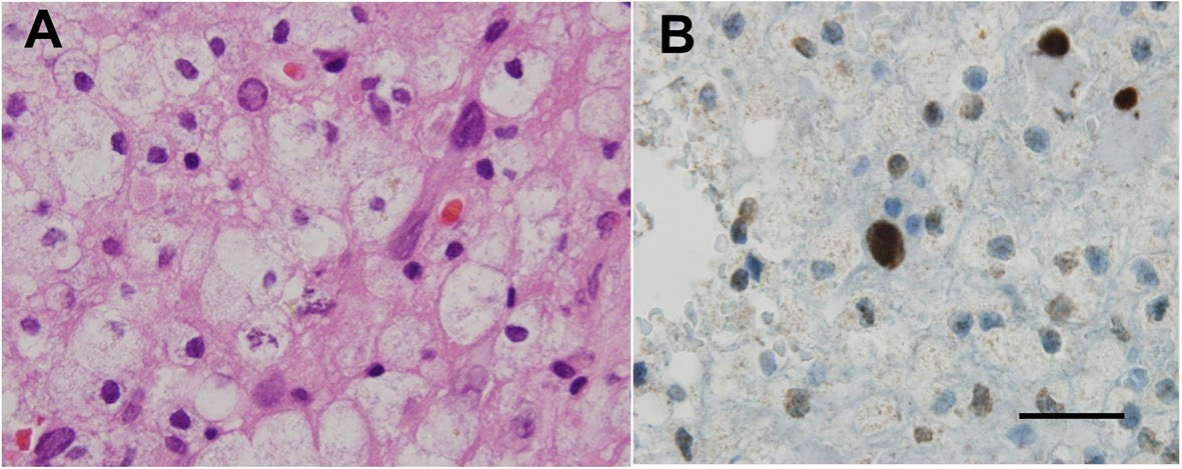
**A** Histopathology (hematoxylin & eosin). The figure shows typical demyelinating pattern with “bizarre-looking” astrocytes. High-power view of the eosinophilic nuclear inclusions. **B** Positive immunohistochemical stain (SV40) for polyomavirus

Routine blood tests were normal; except for differential showed mild lymphopenia (Table 1). Vasculitis screening, HIV, tuberculosis quantiferon, syphilis and toxoplasma screening were negative. Brain MRI showed diffuse subcortical changes, and the white matter lesions were gradually aggravated with the progress of the disease (Fig. 1). T-cell subset analysis showed CD4+ T-cell count 286/μL↓ (410–1590/μL) and CD4^+^/CD8^+^ = 0.66↓. CSF metagenomics sequencing was performed, the number of standardized strict mapping reads of JC polyomavirus was 78. A stereotactic biopsy, that included the right frontal lobe brain parenchyma and periventricular region, showed astrogliosis with foci of histiocytic infiltration, eosinophilic nuclear inclusions, and severe demyelination with numerous macrophages. Immunostaining for SV40 were positive in inclusion-bearing cells (Fig. 2). This was consistent with active PML.

**Table 1:** Absolute numbers of lymphocytes from admission to 5 months of disease evolution

Date	lymphocytic counts	Reference range, Adults
3-26-2020	0.79 × 10^9^/L	1.1--3.2 × 10^9^/L
5-18-2020	0.67 × 10^9^/L	1.1--3.2 × 10^9^/L
5-25-2020	0.84 × 10^9^/L	1.1--3.2 × 10^9^/L
6-13-2020	0.95 × 10^9^/L	1.1--3.2 × 10^9^/L
7-01-2020	0.98 × 10^9^/L	1.1--3.2 × 10^9^/L
7-14-2020	0.91 × 10^9^/L	1.1--3.2 × 10^9^/L
7-23-2020	1.12 × 10^9^/L	1.1--3.2 × 10^9^/L

**Fig. 3:**
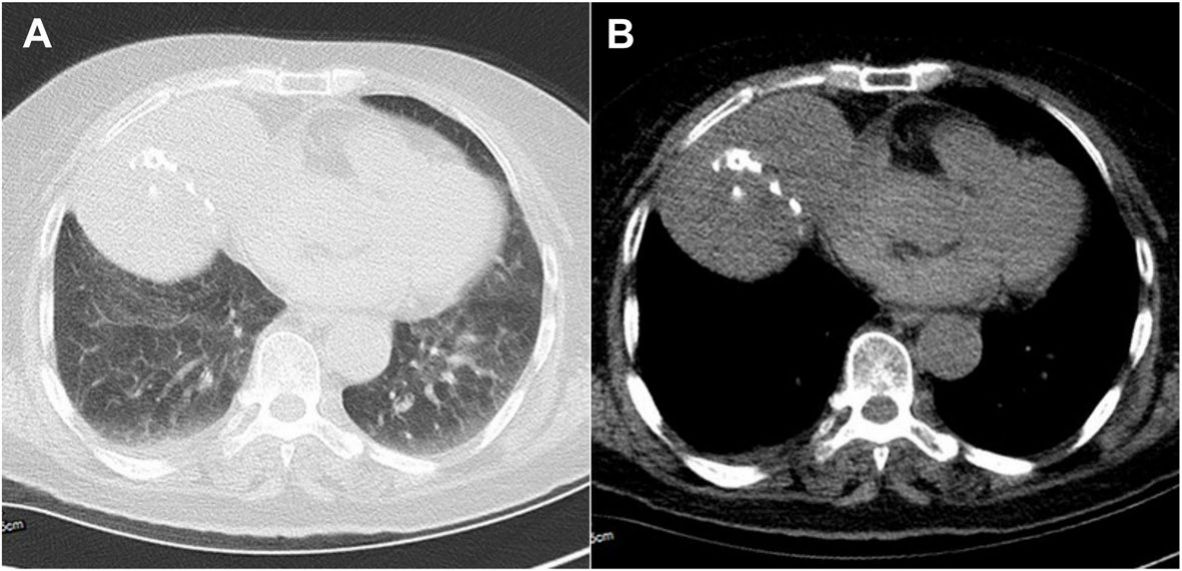
Computerized tomogram images of the chest. Axial views showing a large teratoma with some calcifications. **A** Pulmonary window; **B** Mediastinal window

She was started to take mirtazapine orally and received intravenous immunoglobulin for 5 consecutive days. Routine blood tests show that the lymphocyte was normal and the patient’s condition was relatively stable and approved to be discharged from hospital. On our last contact with the family, the patient was admitted to a local hospital where she received supportive treatment after developing a dense acroparalysis, she continued to deteriorate and died of the lung infection 10 months after first presentation.

## Discussion and conclusions

The clinical presentation of PML is extremely varied, with neurological and psychiatric symptoms dependent on the location primarily affected developing over weeks. The patient had typical onset form, neurological symptoms, history of hypertension, and imaging showed extensive high signal intensity on both T2-FLAIR and DWI. Based on the neurological symptoms and high signal intensity on both T2-FLAIR and DWI, she was diagnosed as cerebral infarction in the local hospital. But the symptoms gradually worsened and the white matter lesions gradually aggravated. In the following examine, JCV virus was detected in CSF through high-throughput detection, and SV40 virus was detected in further brain tissue biopsy. According to the diagnostic criteria previously published by the American academy of neurology (AAN) [9], the patient was diagnosed as PML. In patients with PML, diagnosis is generally supported by characteristic changes at CNS imaging. On MRI, PML lesions are usually hypointense on T1 and hyperintense on T2-FLAIR and DWI, which often presents with ischemic cerebrovascular disease. So we should be careful to identify the localization and qualitative diagnosis, to prevent misdiagnosis.

The patient’s medical history mentioned a large asymptomatic mediastinal teratoma. In our opinion, this case represents mediastinal teratoma associated PML, since other immunosuppressive medications or coexisting medical conditions were absent. The thymus is responsible for the maturation of lymphoid precursors into T cells, and is necessary to establish the T cell pool during prenatal and early postnatal life in humans [10]. Although the ability of thymus generating T cells decreases with aging, the thymus still serves as the site of T-cell differentiation and maturation throughout life [11].

Both longitudinal and cross-sectional studies have revealed multiple immune alterations of the CD4+ and CD8+ T cell compartments in thymectomized subjects [12]. These exacerbated alterations are the likely consequence of the strong and persistent mobilization of cellular immunity by this virus in the absence of adequate T cell renewal capacity due to thymectomy, eventually resulting in a premature exhaustion of T cell resources [13]. So, Lymphopenia may be due to the compression of thymus gland by anterior mediastinal teratoma. Our limitation is that we were not able to confirm the suspected diagnosis of teratoma with histopathological findings in this case. Our patient was treated with mirtazapine and immunoglobulin. Mirtazapine could block glial cell infection via the 5HT2a receptor [14], and immunoglobulin could improve the lymphopenia. The lymphopenia was corrected after intravenous administration of immunoglobulin.

To our knowledge, this is the first reported case of mediastinal teratoma associated PML in a patient without severe lymphocytopenia. This case highlights several issues. First, anterior mediastinal teratoma might induce lymphopenia and trigger PML. Second, for patients with subacute generalized leukoencephalopathy, even if there is no obvious immunosuppressive disease and inducement, we should consider the possibility of this disease. Third, when there is no obvious cause of PML, it is important to search for an underlying immunological disorder.

## Data Availability

The datasets used and analyzed during the current study are available from the corresponding author on reasonable request.

## Abbreviations

CSF: Cerebrospinal fluid
DNA: Desoxyribonucleic acid
HIV: Human immunodeficiency virus
JCV: JC polyomavirus
MRI: Magnet resonance imaging
PCR: Polymerase chain reaction
PML: Progressive multifocal leukoencephalopathy
SV40: Simian virus 40
